# Assessment of runs of homozygosity islands and estimates of genomic inbreeding in Gyr (*Bos indicus*) dairy cattle

**DOI:** 10.1186/s12864-017-4365-3

**Published:** 2018-01-09

**Authors:** Elisa Peripolli, Nedenia Bonvino Stafuzza, Danísio Prado Munari, André Luís Ferreira Lima, Renato Irgang, Marco Antonio Machado, João Cláudio do Carmo Panetto, Ricardo Vieira Ventura, Fernando Baldi, Marcos Vinícius Gualberto Barbosa da Silva

**Affiliations:** 1 Faculdade de Ciências Agrárias e Veterinárias, Departamento de Zootecnia, UNESP Univ Estadual Paulista Júlio de Mesquita Filho, Jaboticabal, 14884-900 Brazil; 2 Faculdade de Ciências Agrárias e Veterinárias, Departamento de Ciências Exatas, UNESP Univ Estadual Paulista Júlio de Mesquita Filho, Jaboticabal, 14884-900 Brazil; 3 Conselho Nacional de Desenvolvimento Científico e Tecnológico (CNPQ), Lago Sul, 71605-001 Brazil; 4 Centro de Ciências Agrárias, Departamento de Zootecnia e Desenvolvimento Rural, Universidade Federal de Santa Catarina, Florianópolis, 88034-000 Brazil; 5 Embrapa Gado de Leite, Juiz de Fora, 36038-330 Brazil; 6 Faculdade de Zootecnia e Engenharia de Alimentos, Universidade de São Paulo, Pirassununga, 13635-900 Brazil; 7 Beef Improvement Opportunities, Elora, ON N0B 1S0 Canada; 8 University of Guelph, Centre for Genetic Improvement of Livestock, ABScBG, Guelph, N1G 2W1 Canada

**Keywords:** *Bos indicus*, Dairy traits, Inbreeding coefficients, ROH islands

## Abstract

**Background:**

Runs of homozygosity (ROH) are continuous homozygous segments of the DNA sequence. They have been applied to quantify individual autozygosity and used as a potential inbreeding measure in livestock species. The aim of the present study was (i) to investigate genome-wide autozygosity to identify and characterize ROH patterns in Gyr dairy cattle genome; (ii) identify ROH islands for gene content and enrichment in segments shared by more than 50% of the samples, and (iii) compare estimates of molecular inbreeding calculated from ROH (F_ROH_), genomic relationship matrix approach (F_GRM_) and based on the observed versus expected number of homozygous genotypes (F_HOM_), and from pedigree-based coefficient (F_PED_).

**Results:**

ROH were identified in all animals, with an average number of 55.12 ± 10.37 segments and a mean length of 3.17 Mb. Short segments (ROH_1–2 Mb_) were abundant through the genomes, which accounted for 60% of all segments identified, even though the proportion of the genome covered by them was relatively small. The findings obtained in this study suggest that on average 7.01% (175.28 Mb) of the genome of this population is autozygous. Overlapping ROH were evident across the genomes and 14 regions were identified with ROH frequencies exceeding 50% of the whole population. Genes associated with lactation (*TRAPPC9*), milk yield and composition *(IRS2* and *ANG*), and heat adaptation (*HSF1*, *HSPB1,* and *HSPE1*), were identified. Inbreeding coefficients were estimated through the application of F_ROH_, F_GRM_, F_HOM_, and F_PED_ approaches. F_PED_ estimates ranged from 0.00 to 0.327 and F_ROH_ from 0.001 to 0.201. Low to moderate correlations were observed between F_PED_-F_ROH_ and F_GRM_-F_ROH_, with values ranging from −0.11 to 0.51. Low to high correlations were observed between F_ROH_-F_HOM_ and moderate between F_PED_-F_HOM_ and F_GRM_-F_HOM_. Correlations between F_ROH_ from different lengths and F_PED_ gradually increased with ROH length.

**Conclusions:**

Genes inside ROH islands suggest a strong selection for dairy traits and enrichment for Gyr cattle environmental adaptation. Furthermore, low F_PED-_F_ROH_ correlations for small segments indicate that F_PED_ estimates are not the most suitable method to capture ancient inbreeding. The existence of a moderate correlation between larger ROH indicates that F_ROH_ can be used as an alternative to inbreeding estimates in the absence of pedigree records.

**Electronic supplementary material:**

The online version of this article (10.1186/s12864-017-4365-3) contains supplementary material, which is available to authorized users.

## Background

Autozygosity occurs when chromosomal segments arising from a common ancestor are identical by descent (IBD) and inherited from both parents on to the offspring genome [1]. This pattern of inheritance gives rise to continuous IBD homozygous segments characterized as runs of homozygosity (ROH) [2], which can be a consequence of several population phenomena [3]. The development of high-density SNP arrays to scan the genome for ROH has been proposed as a useful method to distinguish non-autozygotic segments that are identical by state (IBS) from those autozygotic and IBD [4].

As the occurrence of ROH tend to be revealed in the genome, its identification and characterization can provide an insight into how population structure and demography have evolved over time [5–7]. ROH can disclose the genetic relationships among individuals, estimating with a high accuracy the autozygosity at the individual and population levels [8–11] and can elucidate about selection pressure events [10, 12, 13]. As the expected length of the autozygous segment follows an exponential distribution with mean equal to 1/2*g* morgans, where *g* is equal to the number of generations since the common ancestor, the number of generations from the selection events can be inferred from the length and frequency of ROH [4].

The autozygosity based on ROH can help to improve the understanding of genetic selection process of quantitative traits as the selection is one of the main forces that tend to print homozygous stretches on the genome [14]. According to Zhang et al. [13], ROH patterns are not randomly distributed across the genomes, and ROH islands are seen to be distributed and shared among individuals, which is likely the result of selection events. Therefore, ROH can be used to explore signatures of selection [12, 14], since genomic regions sharing ROH potentially contain alleles associated with genetic improvement in livestock [6] and are of interest for breeding programs [14]. ROH can also be an accurate estimator of inbreeding considering that high levels of inbreeding increase the frequency of homozygous alleles [10].

Studies have considered pedigree-based estimates of inbreeding (F_PED_) since Wright [15], although the availability of whole-genome marker panels has widespread the use of genomic information in animal breeding [16]. Pedigree-based relatedness is calculated from statistical expectations of the probable proportion of genomic identity by descent, while genotype-based estimates show the current relatedness among individuals [17]. Molecular approaches based on inbreeding coefficient estimates derived from ROH (F_ROH_) and based on genomic relationship matrix (F_GRM_) [18] are meaningfulness to avoid drawbacks of using pedigrees to analyze inbreeding. F_ROH_ are worth to estimate genome-wide autozygosity as it captures the influence of relatedness among founders. F_ROH_ also takes into account the stochastic nature of recombination and mutations loads [19], and the potential bias resulting from selection [20] as well.

The first Gyr (*Bos primigenius indicus*) animals in Brazil had arrived in 1912, and most of the bulls were imported between 1914 and 1921, being then incorporated in crosses [21]. Those imported animals were first consumed for beef cattle purpose, and some breeders started to use them for milk production. Gyr animals have been intensely applied as the basis for crosses with taurine dairy breeds due to its rusticity and greater tolerance to the tropical environment [22]. The mating between imported animals invariably led to a steep increase in inbreeding rate in the population, resulting in genetic gains and fixation of favorable alleles. Over time, the deleterious effects associated with boosted homozygosity arising from inbreeding are predisposed to reduce the genetic gains, implicating in a clear loss of genetic variability (reviewed by Peripolli et al. [23]). Hence, the intense use of founders’ animals to create the first Gyr dairy lines presumably triggered the autozygosity. This outcome is due in part to the inexistence of a breeding program at the time [24], the limited number of animals imported from India and the small number of proven sires mated to disseminate the breed [25]. Therefore, maintaining genetic variability in the Gyr cattle in Brazil is a demanding issue, since Brazil is recognized as a Gyr genetic supplier to some tropical countries that have deficiencies in milk production. Genome-wide autozygosity is an upcoming research area with a growing interest in characterizing and comprehending the mechanisms involved in it, so as to preserve a long-term viability and sustainability of Gyr breeding programs.

The aim of this work was to assess genome-wide autozygosity in Gyr cattle to identify and characterize ROH patterns, as well as to investigate ROH islands for dairy gene content in segments shared by more than 50% of the population. Further, we aimed to compare estimates of molecular inbreeding calculated from F_ROH_, F_GRM_ and based on the observed versus expected number of homozygous genotypes (F_HOM_) with those obtained from F_PED._

## Results

### Genomic distribution of runs of homozygosity

**Fig. 1:**
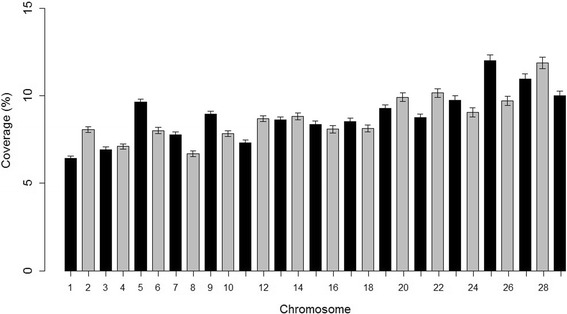
Average percentage of chromosome coverage by runs of homozygosity of minimum length of 1 Mb. The error bars indicate the standard error

ROH were identified in all 2908 individuals, totaling 161,362 homozygous segments among overall samples. On an individual animal basis, the average number of ROH per animal was 55.12 ± 10.37, with values ranging from 17 to 121. The mean ROH length was 3.17 Mb and the longest segment was 108.97 Mb in length (33,050 SNPs) found on BTA8. The number of ROH per chromosome was greater for BTA5 (10,670 segments) and tended to decrease with chromosome length. The major fraction of chromosome residing in ROH was found on BTA25 (11.98% of chromosomal length in a ROH) (Fig. 1).

Descriptive statistic of ROH number and length by classes is given in Table 1. The total length of ROH for Gyr is composed mostly of a high number of shorter segments (ROH_1–2 Mb_). These segments accounted for approximately 60% of all ROH detected, which contributed, however, for less than 25% of the cumulative ROH length. While shorter ROH were abundant throughout the genome, the proportion of the genome covered by them was relatively small. In contrast, larger ROH (ROH_>16 Mb_) were at least twenty-five fold less abundant than shorter ROH (ROH_1–2 Mb_) and still covered a higher proportion of the genome than small and medium ROH.

**Table 1:** Descriptive statistics of runs of homozygosity number (*n* ROH) and length (in Mb) by ROH length class (ROH_1 − 2 MB_, ROH_2 − 4 MB_, ROH_4 − 8 MB_, ROH_8–16 MB_, and ROH_>16 Mb_)

Class	*n* ROH	Percent	Mean length	Standard deviation	Genome coverage (%)
ROH_1–2 Mb_	95,892	59.42	1.34	0.27	1.77
ROH_2–4 Mb_	35,395	21.93	2.77	0.55	1.34
ROH_4–8 Mb_	17,843	11.05	5.54	1.12	1.36
ROH_8–16 Mb_	8518	5.27	10.98	2.17	1.46
ROH_>16 Mb_	3714	2.30	25.23	10.06	2.33

**Fig. 2:**
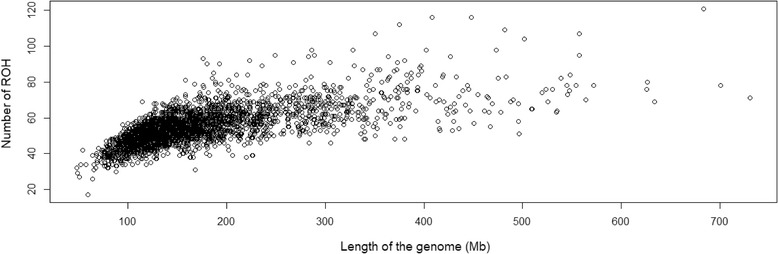
Number of ROH per individual and the length of the genome covered by ROH

The animal displaying the highest autozygosity exhibited a ROH genome coverage encompassing 730.21 Mb of the total autosomal genome extension covered by markers (29.20% of the cattle genome), with 71 ROH ≥ ROH_1–2 Mb_, and a mean ROH length of 10.28 Mb. The least inbred animal exhibited a ROH genome coverage encompassing 48.81 Mb (1.95% of the cattle genome), with 32 ROH ≥ ROH_1–2 Mb_, and a mean ROH length of 1.52 Mb. Differences among animals regarding the number of ROH and the length of the genome covered by them were observed (Fig. 2). The sum of all ROH per animal allowed the estimation of the percentage of the genome that is autozygous and an average value of 7.01% (175.28 Mb) was observed.

### Gene characterization in ROH islands

**Fig. 3:**
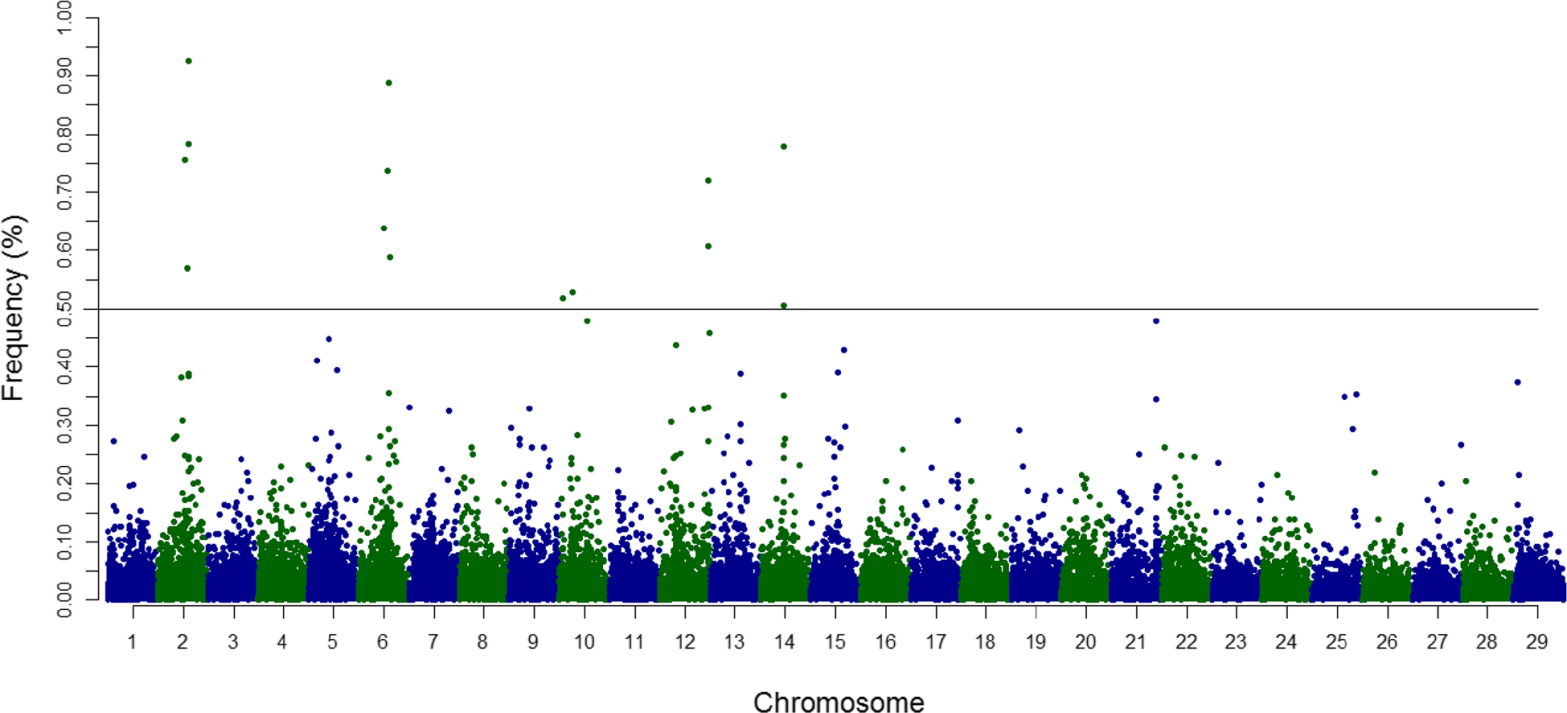
Manhattan plot of the distribution of runs of homozygosity (ROH) islands in the Gyr cattle genome. The X-axis represents the distribution of ROH across the genome, and the Y-axis shows the frequency (%) of overlapping ROH shared among samples

Overlapping ROH were evident across the genome, and their genomic distribution was non-uniform both in length and position across chromosomes. A total of 14 regions with outlying ROH frequencies on BTA2, BTA6, BTA10, BTA12, and BTA14 were identified (Additional file 1). Among the described ROH islands, the strongest pattern was observed on BTA2 (78,394,916:87,587,063 bp), with an overlapping ROH region present in 92% of the samples (Fig. 3). The majority of SNPs within ROH regions showed higher linkage disequilibrium (LD) levels than the estimates obtained for the entire chromosome (Additional file 2).

A relevant number of genes (*n* = 282) inside these ROH islands were observed (Additional file 1), in which several of them play important role in the mammary gland biology and have a prominent importance in milk, dairy traits, and heat adaptation. Gene ontology (GO) and pathway analysis (KEEG) were performed by DAVID tool [26, 27] to obtain a broad functional insight into the set of genes. An enrichment of genes involved in several GO terms (14 molecular functions, 23 biological processes, and seven cellular components) and KEGG pathways was observed (Additional file 3).

### Pedigree and genomic inbreeding

Descriptive statistics for F_PED_ and F_ROH_ coefficients are presented in Table 2. Among F_ROH_ estimates, it can be observed an increase in variation with ROH length, being evidenced by the coefficient of variation (CV).

**Table 2:** Descriptive statistics of the pedigree-based inbreeding coefficient (F_PED_) and genomic inbreeding coefficients based on runs of homozygosity (F_ROH_) for different lenghts (F_ROH1–2 Mb_, F_ROH2–4 Mb_, F_ROH4–8 Mb_, F_ROH8–16 Mb_, and F_ROH > 16 Mb_) for genotyped animals (*n*)

Inbreeding coefficient	Mean	Median	Minimum	Maximum	Coefficient of Variation (%)	*n*
F_PED_	0.019	0.004	0.000	0.327	3.38	2758
F_ROH1–2 Mb_	0.017	0.017	0.006	0.037	20.70	2758
F_ROH2–4 Mb_	0.013	0.013	0.001	0.039	35.30	2757
F_ROH4–8 Mb_	0.013	0.012	0.001	0.063	55.63	2740
F_ROH8–16 Mb_	0.014	0.012	0.003	0.082	73.04	2422
F_ROH > 16 Mb_	0.023	0.016	0.006	0.201	97.75	1533

**Fig. 4:**
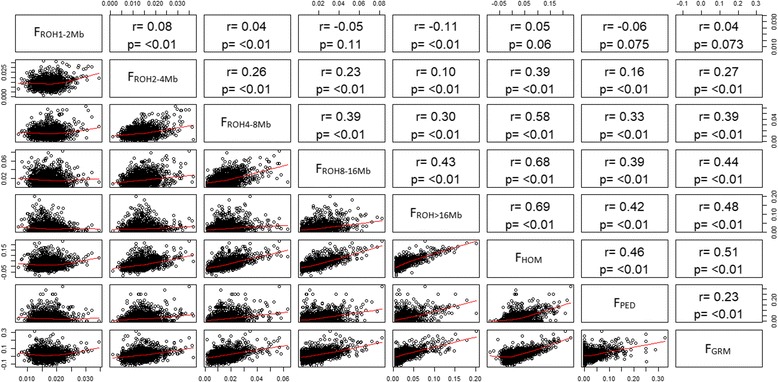
Scatterplots (lower panel) and correlations (upper panel) of genomic inbreeding coefficients F_ROH_ (F_ROH 1–2 Mb_, F_ROH 2–4 Mb_, F_ROH 4–8 Mb_, F_ROH 8–16 Mb_, and F_ROH > 16 Mb_) and F_GRM_, and pedigree-based inbreeding coefficients (F_PED_)

Low to moderate correlations were observed between F_PED_-F_ROH_ and it increased with ROH length (Fig. 4). Additionally, F_PED_ slightly correlated with F_GRM_ (0.23). The correlations between F_GRM-_F_ROH_ were higher than those between F_PED_-F_ROH_ for all ROH classes described. F_HOM_ highly correlated with F_ROH_ over than 4 Mb, F_PED_, and F_GRM_.

**Fig. 5:**
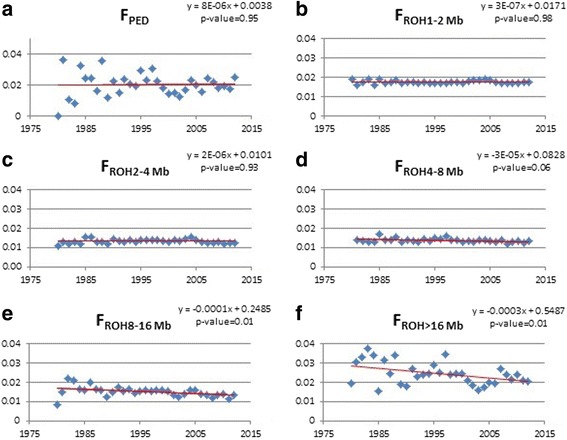
Inbreeding evolution over the past 30 years for pedigree-based inbreeding (F_PED_) and F_ROH_ (F_ROH1–2 Mb_, F_ROH2–4 Mb_, F_ROH4–8 Mb_, F_ROH8–16 Mb_, and F_ROH > 16 Mb_) coefficients. Linear regression (red) in function of the year (x-axis) and inbreeding (y-axis). Each blue dot represents the average F_PED_ and F_ROH_ observed per year

The inbreeding evolution (F_PED_ and F_ROH_) for animals born between 1980 and 2012 is shown in Fig. 5 and the genotyping sampling of animals per inbreeding coefficient in Table 2. The F_PED_ evolution showed a tendency to slightly increase over time (Fig. 5a), while F_ROH_ tended to decrease for segments higher than 4 Mb (Fig. 5d-f).

## Discussion

### Genomic runs of homozygosity patterns

The greatest number of ROH per chromosome was described on BTA5, however, results in taurine breeds [6, 28, 29] have evidenced the highest number of ROH on BTA1. The longest ROH was found on BTA8 with 108.97 Mb in length and similar results on BTA8 were reported by Kim et al. [10] in a contemporary Holstein cow (87.13 Mb) and Mastrangelo et al. [28] in Cinisara cattle breed (112.65 Mb).

The number of generations of inbreeding can be inferred from the extent of ROH since their extension is expected to correlate to ancient and recent inbreeding due to recombination events [1]. Therefore, due to recent inbreeding, ROH are expected to be longer since recombination did not have enough time to break up these IBD segments, while short ROH tend to reflect ancient inbreeding because the segments have been broken down by repeated meiosis [30]. The presence of segments larger than 10 Mb is traceable to inbreeding from recent common ancestors that occurred only five generations ago [4], and 78% of the animals comprised in this study presented at least one homozygous segment extending over 10 Mb. The reflection of a recent parental relatedness for animals with segments longer than 10 Mb was confirmed when analyzing the pedigree back in only two generations, in which animals were seen to be inbred by their grand and great-grandparents.

The highest autozygosity value per animal was similar to those reported in the literature for dairy breeds. Purfield et al. [6] observed that dairy breeds were the most autozygous animals among several studied breeds, and had on average 700.3 Mb of their genome classified as ROH. Mastrangelo et al. [28] observed a close value for the Reggiana dairy breed (725.2 Mb) and also did Szmatoła et al. [31] for Holstein cattle with 25% of their genome located in ROH. It is noteworthy to highlight that Marras et al. [14] described that dairy breeds had a higher sum of all ROH than did beef breeds. The higher autozygosity observed in dairy breeds can be explained by the intense artificial selection and the repeatedly use of superior and proven sires for reproduction by artificial insemination [10]. In the Gyr cattle, it can be attributed to the rapid growth and dissemination of the breed over the last years, in which a small number of proven sires with high breeding value were frequently used [32].

Animals with the same length of the genome covered by ROH displayed a variable number of segments, which is likely a consequence of the distinct distances from the common ancestor, as also described by Mészáros et al. [33]. Overall, the autozygotic proportion of the genome found in this population was considered low given the Gyr dissemination historical process. A similar value was achieved by Marras et al. [14] (7% for Marchigiana beef breed). Gyr cattle presented a lower genome average autozygosity compared to previous studies reported by Ferenčaković et al. [8] (9% for Austrian dual purpose Simmental, Brown Swiss, and Tyrol Grey bulls) and Kim et al. [10] (10% for Holstein cattle), and a higher autozygosity than results obtained by Zavarez et al. [11] (4.58% for Nellore cattle).

### Runs of homozygosity islands and gene functional annotation

The overlapping ROH regions observed across the genome suggest that these regions are likely a sign of ROH islands shared among animals [9]. ROH islands can be defined as genomic regions with reduced genetic diversity and, consequently, high homozygosity around the selected locus that might harbor targets of positive selection and are under strong selective pressure [34]. The strongest ROH island pattern on BTA2 (78,394,916:87,587,063 bp) present in 92% of the samples showed an enrichment of genes involved with the immunity system. Similarly, Marras et al. [14] reported a ROH in 90% of the samples in Piedmontese cattle, although it was located at the beginning of BTA2, closest to the myostatin (*MSTN*) locus. Karimi [12] identified the most common pattern in indicine breeds on BTA21, with a value exceeding 93% of individuals.

The high LD levels found in the majority of SNPs within the ROH islands are not surprisingly since selection in cattle has possibly acted to maintain conserved ROH regions originated from IBD segments. These segments are likely to have experienced fewer recombination events and they are expected to display high levels of LD. Besides, a study on human populations has shown a correlation between extensive LD, locally low recombination rates and high incidence of ROH [2].

Several genomic regions with significant SNPs (−log10(p) > 4) based on the integrated Haplotype Score (iHS) were identified for the Gyr cattle by Utsunomiya et al. [35], using a subset of Gyr animals comprised in this study. Of the significant SNPs, seven of them were located within ROH islands described here on BTA2, BTA6, and BTA10 (Additional file 4).

When analyzing genomic positions of the identified ROH islands, the results pointed out by Szmatoła et al. [31] directly overlaps with some of the islands found in our study, with similar regions identified on BTA2 for Holstein, Polish Red and Limousin breeds, on BTA6 for Polish Red, Limousin and Simmental breeds, and on BTA14 for the Simmental cattle (Additional file 4). The Simmental breed also showed a ROH island on BTA6 located closely to the one described in this study for the Gyr cattle (70,117,799:81,603,050 bp). Karimi [12] and Sölkner et al. [36] study on Brahman, Gyr and Nellore cattle also identified ROH islands in some chromosomes as those described in this study. Although the islands on BTA10 and BTA12 were not found to be located at the same genomic region as in our study, the described island on BTA10 was found closest to ours. ROH islands identified on BTA6 were also described in Italian Holstein cattle [37], dairy and beef breeds [14], and in Tyrol Grey cattle [33], but none of them overlapped with those previously described for the Gyr cattle in this study. It is worth to highlight that BTA6 is well documented to harbor genes that affect milk production traits [38–41], thus, a high autozygosity in chromosomal regions may be an indicator of signatures of selection for dairy traits. Further, ROH islands were found overlapping in cattle breeds selected for different purposes, suggesting that selection pressure can also be undergoing on traits other than those specific to dairy or beef traits.

The GO analyses showed several enriched terms for the ROH gene list. A total of 10 genes were identified related to cell differentiation biological process (GO:0030154), in which we highlight the *TRAPPC9* (trafficking protein particle complex 9) gene on BTA14. Interestingly, this gene was found to have significant effects on mastitis-related traits in Chinese Holstein herds [42]. Polymorphisms in *TRAPPC9* gene has been associated with milk production traits in Holstein cattle [43]. Jiang et al. [44] observed a higher TRAPPC9 mRNA expression level in the mammary gland of lactating cows than in the other tissues, such as heart, liver, lung, kidney, ovary, uterus, and muscle.

Seven genes identified in ROH islands were related to positive regulation of cell migration (GO:0030335) biological process. Of these, the *IRS2* (insulin receptor substrate 2), *ATP8A1* (ATPase phospholipid transporting 8A1), *GABRG1* (gamma-aminobutyric acid type A receptor gamma1 subunit), and *GABRAG2* (gamma-aminobutyric acid type A receptor gamma2 subunit) genes have been previously associated with dairy traits. The *IRS2* gene on BTA12 encodes the insulin receptor substrate 2, a cytoplasmic signaling molecule that mediates effects of insulin, insulin-like growth factor 1 and other cytokines (provided by RefSeq, Jul 2008). Insulin infusion has been shown to increase milk and protein yields, and reduce milk fat content and yield in lactating goats. It also decreased net uptake of C10:0, C14:0, C16:0, trans-C16:1 and >C18:0 fatty acids, and increased mammary blood flow by 42% [45]. The *ATP8A1*, *GABRG1*, and *GABRAG2* genes on BTA6 laid within the region with highest iHS score as reported by Hayes et al. [46] in Norwegian Red cattle, a breed which has been intensely selected for milk production.

The nuclear stress granule (GO:0097165) cellular component was substantially enriched (*p* ≤ 0.05), which contains the *HSF1* (heat shock transcription factor 1) gene on BTA14. This gene encodes a heat-shock transcription factor, and its transcription is rapidly induced after heat stress (provided by RefSeq, Jul 2008). Heat shock transcription factors and heat shock proteins (HSP) play a crucial role in environmental stress adaptation and thermal balance since it allows cells to adapt to gradual environmental changes, being an immunoregulatory agent upon controlling the balance between survival and an effective immune system in order to adjust to stress [47]. Kumar et al. [48] observed a higher abundance of *HSP* family genes during summer and winter compared to the mid-spring season in *Bos indicus* cattle and Murrah buffaloes, and the magnitude of increase was higher during summer as compared to winter. Among their findings, a significantly (*p* ≤ 0.001) higher HSF1 mRNA expression during the summer as compared to the mid-spring season was also observed. These findings are consistent with the zebu cattle adaptation traits, in which we highlight its greater ability to tolerate poor feed and inconsistent climate. Li et al. [49] identified polymorphisms in *HSF1* gene associated with thermal tolerance in Holstein cattle. In addition to the HSF1 gene, other heat shock genes were found within a ROH island on BTA2, such as *HSPD1* (heat shock protein family D (Hsp60) member 1) and *HSPE1* (heat shock protein family E (Hsp10) member 1).

We also encountered a number of genes within ROH islands that have been reported to have a prominent importance in milk-related traits on BTA2 (*STAT1* and *INSIG2* genes) [50, 51], BTA10 (*ANG* gene) [52] and BTA14 (*EEF1D*, *CRH*, *DGAT1,* and *CYP11B1* genes) [53–60]. In addition, mammary gland development-related genes were also described on BTA6 (*IGFBP7* gene) [61, 62] and BTA14 (*EEF1D* gene) [44, 63].

A total of seven KEGG pathways were identified as being enriched (*p* ≤ 0.05) and the GABAergic synapse (bta04727) was the most significant (*p* < 0.001) KEGG pathway found (Additional file 3). Gamma-aminobutyric acid (GABA) is an inhibitory neurotransmitter in the mammalian central nervous system and the GABAergic synapse pathway has been associated with animal feed intake and weight gain [64]. Among the others KEGG pathways identified, the ones related to environmental information processing were highlighted, such as neuroactive ligand-receptor interaction (bta04080), PI3K-Akt signaling pathway (bta04151), and AMPK signaling pathway (bta04152) with 11, 10, and 5 genes identified within ROH islands, respectively. PI3K-Akt signaling pathway regulates key cellular functions such as transcription, translation, growth, proliferation, and survival. This pathway has been associated with prolactin signaling, mammary development, and involution in Holstein-Friesian and Jersey breeds [65]. AMPK signaling pathway acts as a sensor of cellular energy status leading to a concomitant inhibition of energy-consuming biosynthetic pathways and activation of ATP-producing catabolic pathways.

Instead of being randomly distributed across the genomes, ROH patterns were seen clustering in specific genomic regions among individuals. These regions were screened for genes under selection and several ROH islands harboring dairy-related genes have been identified, suggesting a directional selection for milk and mastitis-related traits, mammary gland development, and environmental adaptation traits. Surprisingly, BTA14 has shown an enrichment of genes affecting traits of interest for dairy breeders. BTA2 and BTA6 also have shown ROH islands previously described in the literature, and these chromosomes along with BTA10 also revealed signatures of selection previously identified for the Gyr breed [33]. These findings suggest that these chromosomes are likely to contain traces of selection since ROH patterns are not expected to be randomly distributed over the genomes [13]. Also, they evidenced that ROH can reveal signatures of selection since ROH islands described in here corroborated with footprints of recent positive selection previously described for the Gyr cattle [35].

### Inbreeding coefficients

The higher the CV was, the greater the differences between the mean and median were for each F_ROH_ length (Table 2). Thus, given the dissimilarity among the CV, it is assumed that the mean should not be used as the best measurement of central tendency, indicating that the median should be applied instead for F_PED_ and F_ROH_ coefficients. The average F_PED_ and F_ROH_ were low for the Gyr cattle, and the F_PED_ estimate was lower than those reported by Reis Filho et al. [24] and Santana Junior et al. [32] for Brazilian Gyr cattle, with values of 2.82 and 1.92%, respectively.

The age of inbreeding can be defined as the distance with the common ancestor and there is an approximate correlation with the length of the ROH [4, 66]. Under the assumption that 1 cM equals to 1 Mb [4], calculated F_ROH_ are expected to correspond to the reference ancestral population dating 50 (F_ROH1–2 Mb_), 20 (F_ROH2–4 Mb_), 12.5 (_FROH4–8 Mb_), 6 (F_ROH8–16 Mb_), and 3 (F_ROH > 16 Mb_) generations ago. Zavarez et al. [11] observed that incomplete pedigree fails to capture remote inbreeding and estimates based on F_PED_ are only comparable with F_ROH_ calculated over large ROH. Thus, given the average pedigree depth of three generations, F_PED_ estimate should be comparable with F_ROH > 16 Mb_. The variation between these two estimates can be attributed to the fact that F_PED_ assumes that the entire genome does not undergo selection [20] and recombination events, therefore, it does not take into account potential bias from these events [67]. In addition, it should be underlined that pedigree relatedness is estimated from statistical expectations of the probable IBD genomic proportion, whereas genotype-based estimates show the actual relatedness among individuals [17] and can provide greater accuracy on relatedness.

The increasing correlation between F_PED_-F_ROH_ with ROH length may be explained by considering that ROH reflect both past and recent relatedness and that F_PED_ estimates are based on pedigree records which may not extend back many generations [9, 14]. When longer ROH reflecting recent relatedness are considered to calculate F_ROH_, the F_PED_-F_ROH_ correlation tends to be higher [14, 68]. Several authors have described a high F_PED_-F_ROH_ correlation when a deeper number of described generations are available in the pedigree [6, 8, 9, 14, 29], suggesting that the correlation between these parameters increases with pedigree deep. Ferenčaković et al. [8, 9] observed F_PED_-F_ROH_ correlations values ranging from 0.61 to 0.67 and 0.50 to 0.72, respectively, for pedigrees with more than five generations. Purfield et al. [6] used a complete generation equivalents higher than six and obtained F_PED_-F_ROH_ correlations of 0.73 for ROH > 10 Mb and 0.71 for ROH > 1 Mb, both with the reduced panel. Marras et al. [14] observed high F_PED_-F_ROH_ correlations using pedigree with four, seven and ten generations, with values ranging from 0.56 to 0.74. Gurgul et al. [29] also reported the highest F_PED_-F_ROH_ correlation for animals with seven complete generations of pedigree data, with an average value of 0.45. In the present study, a small number of generations were available to estimate F_PED_, which may have introduced biased F_PED_ values as the pedigree was not able to cover ancient relatedness.

The slight correlation between F_PED_-F_GRM_ concurs with the results obtained by Pryce et al. [69]. VanRaden et al. [70] reported higher correlations for Holstein (0.59), Jersey (0.68), and Brown Swiss (0.61) animals. Hayes and Goddard [71] also obtained higher correlations for Australian Angus bulls (0.69). Lower correlations between these estimates were reported by Marras et al. [14], Gurgul et al. [29], and Zhang et al. [72]. In the dairy industry, genomic inbreeding coefficients of genotyped animals are commonly calculated from F_GRM_ [73]_._ Two out of three reasons hypothesized by Pryce et al. [69] might explain the poor correlation found out in our study: (i) F_GRM_ is strongly dependent on allele frequencies, and population with divergent allele frequencies can lead to misleading IBD results; and (ii) pedigree completeness.

It is well addressed in the literature that incomplete pedigree information reduces estimates of inbreeding and leads to underestimated values [74, 75], as well as missing or incorrect pedigree information. Hence, accurate estimates of F_PED_ depend on a well-structured pedigree dataset. When analyzing the Gyr pedigree structure, it was observed that 72.96% of the animals available in the pedigree dataset had both known sire and dam information, and 3.52% had only known sire and 1.16% known dam information. On the basis of the results, F_PED_ estimate might have been underestimated as well as its correlations with other inbreeding measurements due in part to the poor pedigree depth and pedigree incompleteness.

Several studies also have found a low to moderate F_GRM-_F_ROH_ correlation for dairy breeds [14, 28]. In Holstein cattle, moderate to high correlations were described by Bjelland et al. [73] (0.81). Pryce et al. [69] observed a correlation of 0.62 in Holstein and Jersey populations, and Zavarez et al. [11] correlations ranging from 0.41 to 0.74 in Nellore cattle based on ROH of different minimum lengths. Further, the moderate to high correlations between F_ROH_ and the two other estimates of genomic inbreeding (F_GRM_ and F_HOM_) suggest that the proportion of the genome in ROH can be an accurate estimator of the IBD genomic proportion.

The inbreeding evolution illustration (Fig. 5) stress out a significant (*p* < 0.01) decline in F_ROH > 8–16 Mb_ and F_ROH > 16 Mb_, and it is worth to highlight that these coefficients reflect an inbreeding up to six and just three generations ago, respectively. The F_ROH > 8–16 Mb_ and F_ROH > 16 Mb_ coefficients reduction since the 80’s happen together with the creation of the Brazilian Dairy Gyr Breeding Program (PNMGL) and the implementation of the Gyr progeny testing, both in 1985. Probably, these facts suggest that different proven sires from divergent lines started to be incorporated into the population, and previously closed herds started to make use of these genetically evaluated sires in their breeding programs. Mating between herds increased after 2002, a fact that may have strongly contributed to reducing the average inbreeding by increasing the genetic exchange [32]. Additionally, Santana Junior et al. [32] reported that the degree of nonrandom mating was close to zero at the end of the last decade, indicating that better mating decisions were taken by the breeders to avoid mating between relatives, changing the mating policy and decreasing the genomic inbreeding level in these populations over time.

These findings reinforce the importance of effective breeding programs for maintaining genetic diversity and suitable inbreeding levels, contributing to a better understanding of the population structure and providing the basis to overcome challenges. Given the Gyr breed growth background, in which a small number of founder animals was imported to Brazil to disseminate the breed, information regarding genetic diversity within the Gyr cattle is therefore essential for genetic improvement and conservation programs.

## Conclusions

Despite the reduced genetic basis and the limited number of animals imported to form the first Gyr dairy lines, the autozygotic proportion of the genome was considerably low in this population. Hence, maintaining a low autozygosity is crucial in cattle breeding populations, avoiding inbreeding depression [76] and reduced response in breeding programs [77]. Several common ROH islands have been found in the Gyr genome, suggesting that ROH might be used to identify genomic regions under selection signatures [78, 79]. Common islands on BTA2 and BTA14 are supposed to be a sign of strong selection for dairy and environmental adaptation traits as several genes associated with them were identified. Low correlations between F_PED-_F_ROH_ may be partly due to the relatively shallow depth of the pedigree, indicating that F_PED_ is not the most suitable method to capture ancient inbreeding. The existence of moderate to high correlations between F_ROH_ and other genomic inbreeding measures suggests that the levels of autozygosity derived from ROH can be used as an accurate estimator of individual inbreeding levels [6, 8, 29, 73]. In addition, when analyzing the inbreeding evolution for the past 30 years, it can be seen a clear decay in F_ROH_ for segments higher than 4 Mb, reinforcing the importance of effective breeding programs and mating management. Our findings contribute to the understanding of the inbreeding effects when assessing genome-wide autozygosity, and how selection can shape the distribution of ROH islands in the cattle genome. Hence, this approach may contribute to comprehend the evolutionary process of the Gyr breed, i.e. selection and domestication process [80, 81], and provide the basis to overcome future challenges.

## Methods

### Animals and genotyping

The animals used in this study comprise the progeny test program from the National Program for Improvement of Dairy Gir (PNMGL), headed by Embrapa Dairy Cattle (Juiz de Fora, Minas Gerais, Brazil) in cooperation with the Brazilian Association of Dairy Gyr Breeders (ABCGIL) and the Brazilian Association of Zebu Breeders (ABCZ). The objective of the program is to promote the genetic improvement of the Gyr dairy cattle, through the identification and selection of genetically superior bulls for fat, protein and total solids in milk, as well as traits associated with animal conformation and management.

A total of 19 dams and 563 sires born between 1964 and 2013 were genotyped with the BovineHD BeadChip (Illumina Inc., San Diego, CA, USA), containing 777,962 markers; 1664 dams with the BovineSNP50 BeadChip (Illumina Inc., San Diego, CA, USA), that contains 54,609 SNP; and 662 dams with the GGP-LD *Indicus* BeadChip (GeneSeek® Genomic Profiler *Indicus* 30 K), that contains 27,533 markers.

Imputation was implemented using the FIMPUTE 2.2 software [82], and lower density panels were imputed to the HD level. Imputation accuracy was 0.99, in accordance with the results presented by Boisin et al. [83] using the same population (0.98). SNPs unsigned to any chromosome and mapped to sexual chromosomes were removed from the dataset. The animals genotyped with the BovineHD BeadChip (Illumina Inc., San Diego, CA, USA) were used as reference population for imputation. The missing genotypes were imputed in the reference population and all the markers were retained. Prior imputation, samples were edited for call rate (< 90%). After editing the reference and imputed genotypes, a total of 2908 animals and 735,236 SNPs were retained for the analyses.

### Runs of homozygosity

ROH were identified in every individual using PLINK v1.90 [84]. The PLINK software uses a sliding window of a specified length or number of homozygous SNPs to scan along each individual’s genotype at each SNP marker position to detect homozygous segments [4]. The parameters and thresholds applied to define a ROH were (i) a sliding window of 50 SNPs across the genome; (ii) the proportion of homozygous overlapping windows was 0.05; (iii) the minimum number of consecutive SNPs included in a ROH was 100; (iv) the minimum length of a ROH was set to 1 Mb; (v) the maximum gap between consecutive homozygous SNPs was 500 kb; (vi) a density of one SNP per 50 kb; and (vii) a maximum of five SNPs with missing genotypes and up to one heterozygous genotype were allowed in a ROH. The ROH were defined by a minimum of 1 Mb in length to avoid short and common ROH that occur throughout the genome due to LD [6]. ROH were classified into five length classes: 1–2, 2–4, 4–8, 8–16, and >16 Mb, identified as ROH_1–2 Mb_, ROH_2–4 Mb_, ROH_4–8 Mb_, ROH_8–16 Mb_, and ROH_>16 Mb,_ respectively.

### Pedigree and genomic inbreeding coefficients

Four types of inbreeding coefficients (F_PED_, F_ROH_, F_GRM_, and F_HOM_) were taken into account. Pedigree-based inbreeding coefficients (F_PED_) were estimated for all animals using pedigree records from a dataset containing 101,351 animals born between 1946 and 2015. The pedigree data was provided by Embrapa Dairy Cattle (Juiz de Fora, Minas Gerais, Brazil). The average pedigree depth was approximately three generations ranging from 0 to 7.85. The F_PED_ was estimated through the software INBUPGF90 [85]. Genomic inbreeding coefficients based on ROH (F_ROH_) were estimated for each animal according to McQuillan et al. [86]:

$$ {F}_{ROH}=\frac{\sum \limits_{j=1}^n{L}_{ROH j}}{L_{total}} $$

where L_ROHj_ is the length of ROH_j_, and L_total_ is the total size of the autosomes covered by markers. L_total_ was taken to be 2,510,605,962 bp, based on the consensus map. For each animal F_ROH_ (F_ROH1–2 Mb_, F_ROH2–4 Mb_, F_ROH4–8 Mb_, F_ROH8–16 Mb_, and F_ROH > 16 Mb_) was calculated based on ROH distribution of five minimum different lengths (ROH_j_): 1–2, 2–4, 4–8, 8–16, and >16 Mb, respectively. A second measure of genomic inbreeding was calculated from a Genomic relationship matrix (G) and was denoted as F_GRM_. The G matrix was calculated according to the method described by VanRaden et al. [70] using the following formula:

$$ G=\frac{ZZ^{\prime }}{2\sum \limits_{i=1}^n{P}_i\left(1-{P}_i\right)} $$

where *Z* is a genotype matrix that contains the 0-2*p* values for homozygotes, 1–2*p* for heterozygotes, and 2-2*p* for opposite homozygotes, where *P*_*i*_ is the reference allele frequency at locus *i*th. The diagonal elements of the matrix G represent the relationship of the animal with itself, thus, it was used to assess the genomic inbreeding coefficient. Inbreeding based on the observed versus expected number of homozygous genotypes (F_HOM_) was calculated in PLINK v1.90 [84] by computing observed and expected autosomal homozygous genotypes counts for each sample, as follows:

$$ {F}_{HOM}=\frac{Observed\ \mathit{\hom}. count- Expected count}{Total observations- Expected count} $$

Spearmann’s correlation coefficients between the inbreeding measures were estimated.

### Gene prospection in shared ROH regions

The homozygous segments shared by more than 50% of the samples were chosen as an indication of possible ROH islands throughout the genome. The --homozyg-group function implemented in PLINK v1.90 [84] was used to assess ROH islands shared among individuals. The Map Viewer of the bovine genome UMD3.1.1 was used for identification of genes in ROH regions, available at “National Center for Biotechnology Information” (NCBI Map Viewer - https://www.ncbi.nlm.nih.gov/mapview/). Database for Annotation, Visualization, and Integrated Discovery (DAVID) v6.8 tool [26, 27] was used to identify significant (*p* ≤ 0.05) Gene Ontology (GO) terms and KEGG (Kyoto Encyclopedia of Genes and Genomes) pathways using the list of genes from ROH islands and the *Bos taurus* annotation file as background.

## Additional files

Additional file 1: Gene content inside runs of homozygosity overlapping regions (ROH Islands). (DOCX 27 kb)

Additional file 2: Mean linkage disequilibrium (LD) estimated considering a physical distance lower than 100 kb between markers for each *Bos taurus* autosome and within each runs of homozygosity island. (DOCX 17 kb)

Additional file 3: Gene Ontology (GO) terms and KEGG pathways enriched (*p* < 0.05) based on runs of homozygosity islands. (DOCX 30 kb)

Additional file 4: Runs of homozygosity islands and signatures of selection located within or closely to those islands observed in the present study. (DOCX 19 kb)

## Abbreviations

ABCGIL: Brazilian Association of Dairy Gyr Breeders
ABCZ: Brazilian Association of Zebu Breeders
CV: Coefficient of variation
DAVID: Database for Annotation, Visualization, and Integrated Discovery
F_GRM_: Genomic relationship matrix-based estimates of inbreeding
F_HOM_: Inbreeding estimate based on observed and expected autosomal homozygous genotypes
F_PED_: Pedigree-based estimates of inbreeding
F_ROH_: ROH-based estimates of inbreeding
G: Genomic relationship matrix
GO: Gene Ontology
IBD: Identical by descent
IBS: Identical by state
KEGG: Kyoto Encyclopedia of Genes and Genomes
LD: Linkage disequilibrium
PNMGL: National Program for Improvement of Dairy Gir
ROH: Runs of homozygosity
