# Supplementary material for: Assessment of runs of homozygosity islands and estimates of genomic inbreeding in Gyr (Bos indicus) dairy cattle
Source: BMC Genomics. 2018 Jan 9;19:34. doi: 10.1186/s12864-017-4365-3 (PMC5759835; doi:10.1186/s12864-017-4365-3)
Supplement: Supplementary file 1 — Gene content inside runs of homozygosity overlapping regions (ROH Islands). (DOCX 27 kb) [file 12864_2017_4365_MOESM1_ESM.docx]

| Additional file 1 - Gene content inside runs of homozygosity overlapping regions (ROH Islands) | | | | |
| --- | --- | --- | --- | --- |
| BTA^1^ | ROH Frequency | Physical Position (bp) | Length (bp) | Genes content |
| 2 | 0.755 | 68,748,659:89,063,900 | 20,315,241 | *CCDC93, INSIG2, DBI, SCTR, CFAP221, PTPN4, EPB41L5, RALB, INHBB, GLI2, TFCP2L1, CLASP1, TSN, CNTNAP5, GYPC, STAT1, STAT4, MYO1B, TMEFF2, SLC39A10, DNAH7, CCDC150, PGAP1, SF3B1, HSPD1, HSPE1, MARS2, BOLL, PLCL1, SATB2* |
| 2 | 0.569 | 68,748,659:104,825,968 | 36,077,309 | *DBI, SCTR, INHBB, GLI2, GYPC, STAT1, STAT4, HSPD1, HSPE1, BOLL, AOX1, NDUFB3, CFLAR, CASP8, FZD7, SUMO1, BMPR2, CD28, CTLA4, NDUFS1, CREB1, CRYGD, CRYGC, CRYGB, IDH1, MAP2, MYL1, LANCL1, FN1* |
| 2 | 0.924 | 78,394,916:87,587,063 | 9,192,147 | *GYPC, LOC100295717, GLS, STAT1, STAT4, TRNAC-GCA, MYO1B, TRNAE-UUC, NABP1, SDPR, TMEFF2, LOC785710, SLC39A10, DNAH7, STK17B, LOC531691, CCDC150, GTF3C3, C2H2orf66, PGAP1, ANKRD44, SF3B1, COQ10B, HSPD1, HSPE1, MOB4, RFTN2, MARS2, BOLL, PLCL1* |
| 2 | 0.781 | 81,983,121:87,587,063 | 5,603,942 | *LOC100138726, LOC781256, SLC39A10, LOC104971271, DNAH7, STK17B, LOC101906937, LOC531691, CCDC150, GTF3C3, C2H2orf66, PGAP1, LOC101907169, ANKRD44, LOC104971272, LOC104971273, LOC782417, LOC504995, SF3B1, COQ10B, HSPD1, HSPE1, MOB4, RFTN2, MARS2, BOLL, PLCL1, LOC104971274, LOC104971275* |
| 6 | 0.638 | 58,133,150:59,323,454 | 1,190,304 | *NWD2, LOC101906734, C6H4orf19, TRNAC-GCA, RELL1, LOC783826, PGM2, LOC783708, LOC781379, LOC101906872, TBC1D1, LOC104972736, LOC104972737, LOC104972738, LOC101907152,* |
| 6 | 0.737 | 62,281,712:81,603,050 | 19,321,338 | *ATP8A1, KCTD8, GABRG1, GABRA2, GABRA4, GABRB1, ATP10D, NFXL1, CNGA1, ZAR1, OCIAD1, CWH43, SPATA18, RASL11B, FIP1L1, LNX1, CHIC2, KIT, KDR, SRD5A3, CLOCK, CEP135, KIAA1211, PPAT, PAICS, HOPX, POLR2B, IGFBP7, ADGRL3, TECRL* |
| 6 | 0.887 | 68,338,834:73,220,200 | 4,881,366 | *CNGA1, NIPAL1, TXK, TEC, SLAIN2, ZAR1, FRYL, OCIAD1, OCIAD2, CWH43, LRRC66, SGCB, SPATA18, USP46, RASL11B, SCFD2, FIP1L1, LNX1 CHIC2, PDGFRA, KIT, KDR, SRD5A3, TMEM165,, CLOCK, PDCL2, NMU, EXOC1, CEP135, KIAA1211* |
| 6 | 0.587 | 70,117,799:81,603,050 | 11,485,251 | *RASL11B, SCFD2, FIP1L1, LNX1, CHIC2, GSX2, PDGFRA, KIT, KDR, SRD5A3, TMEM165, CLOCK, PDCL2, EXOC1, NMU, CEP135, KIAA1211, AASDH, PPAT, PAICS, SRP72, ARL9, THEGL, HOPX, SPINK2, NOA1, POLR2B, IGFBP7, ADGRL3, TECRL* |
| 10 | 0.518 | 5,133,564:8,452,227 | 3,318,663 | *THOC3, CPLX2, LOC104973021, HRH2, LOC104973023, SFXN1, DRD1, GCNT4, LOC104973024, LOC104973025, LOC104970646, ANKRD31, POLK, TRNAY-GUA, HMGCR, COL4A3BP, ANKDD1B, POC5, SV2C, IQGAP2, F2RL2, F2R, LOC100296562, F2RL1, S100Z, CRHBP, AGGF1, LOC781720, ZBED3, PDE8B* |
| 10 | 0.529 | 25,895,397:27,374,489 | 1,479,092 | *RPGRIP1, HNRNPC, ZNF219, ARHGEF40, RNASE13, TPPP2, NDRG2, SLC39A2, METTL17, RNASE2, RNASE1, BRB, RNASE1, RNASE6, RNASE4, ANG, ANG2, RNASE12, RNASE11, RNASE10, PNP, TMEM55B, APEX1, OSGEP, KLHL33, CCNB1IP1, TTC5, OR11H4, OR4N4, OR4K14* |
| 12 | 0.720 | 86,889,033:89,989,632 | 3,100,599 | *LOC781180, FAM155A, LIG4, ABHD13, TNFSF13B, MYO16, TRNAY-AUA, LOC104973669, LOC104973672, LOC101905776, LOC101905821, LOC104973673, IRS2, LOC104973674, COL4A1, LOC104973675, COL4A2, LOC104973676, LOC104973677, RAB20, NAXD, CARS2, ANKRD10, ING1, LOC784176, LOC104973678, ARHGEF7, TEX29, LOC101906959* |
| 12 | 0.606 | 86,889,033:89,992,862 | 3,103,829 | *LOC781180, FAM155A, LIG4, ABHD13, TNFSF13B, MYO16, CARS2, TRNAY-AUA, LOC104973669, LOC104973672, LOC101905776, LOC101905821, LOC104973673, IRS2, LOC104973674, COL4A1, LOC104973675, COL4A2,LOC104973676, LOC104973677, RAB20, NAXD, ANKRD10, ING1, LOC784176, ARHGEF7, TEX29, LOC101906959, LOC104973678* |
| 14 | 0.778 | 37,250,059:42,032,707 | 4,782,648 | *EYA1, TRNAC-ACA, MSC, TRPA1, MIR1603, KCNB2, TERF1, SBSPON, LOC101906455, C14H8orf89, RPL7, RDH10, TRNAG-UCC, MIR2284L, STAU2, UBE2W, TCEB1, TMEM70, LY96, TRNAE-UUC, LOC104974051, JPH1, GDAP1, LOC104974053, LOC104974054, PI15, CRISPLD1, HNF4G, LOC104974057, ZFHX4* |
| 14 | 0.505 | 39,495,608:41,685,719 | 2,190,111 | *SLC39A4, CPSF1, DGAT1, HSF1, CYC1, EEF1D, CYP11B1, PTK2, TRAPPC9, TG, MYC, DERL1, HAS2, CEBPD, PRKDC, ATP6V1H, RGS20, MOS, PLAG1, PENK, RAB2A, ASPH,CRH, MYBL1, ARFGEF1* |
| 1 *Bos taurus* autosome (BTA) that presented a frequency of overlapping ROH shared by more than 50% of the animals. | | | | |
