# Supplementary material for: Assessment of runs of homozygosity islands and estimates of genomic inbreeding in Gyr (Bos indicus) dairy cattle
Source: BMC Genomics. 2018 Jan 9;19:34. doi: 10.1186/s12864-017-4365-3 (PMC5759835; doi:10.1186/s12864-017-4365-3)
Supplement: Supplementary file 2 — Mean linkage disequilibrium (LD) estimated considering a physical distance lower than 100 kb between markers for each Bos taurus autosome and within each runs of homozygosity island. (DOCX 17 kb) [file 12864_2017_4365_MOESM2_ESM.docx]

| Additional file 2 – Mean linkage disequilibrium (LD) estimated considering a physical distance lower than 100 kb between markers for each *Bos taurus* autosome and within each runs of homozygosity island. | | | |
| --- | --- | --- | --- |
| BTA^1^ | LD^2^ (BTA) | Physical Position (bp) | LD (ROH Island^3^) |
| 2 | 0.28 | 68,748,659:89,063,900 | 0.33 |
| 2 |  | 68,748,659:104,825,968 | 0.30 |
| 2 |  | 78,394,916:87,587,063 | 0.35 |
| 2 |  | 81,983,121:87,587,063 | 0.39 |
| 6 | 0.27 | 58,133,150:59,323,454 | 0.36 |
| 6 |  | 62,281,712:81,603,050 | 0.34 |
| 6 |  | 68,338,834:73,220,200 | 0.34 |
| 6 |  | 70,117,799:81,603,050 | 0.36 |
| 10 | 0.26 | 5,133,564:8,452,227 | 0.22 |
| 10 |  | 25,895,397:27,374,489 | 0.34 |
| 12 | 0.25 | 86,889,033:89,989,632 | 0.27 |
| 12 |  | 86,889,033:89,992,862 | 0.27 |
| 14 | 0.26 | 37,250,059:42,032,707 | 0.27 |
| 14 |  | 39,495,608:41,685,719 | 0.33 |
| ^1^BTA: *Bos taurus* autosome; ^2^ LD: Linkage Disequilibrium; ^3^Region that presented a frequency of overlapping runs of homozygosity (ROH) shared by more than 50% of the animals. | | | |
