# Supplementary material for: Assessment of runs of homozygosity islands and estimates of genomic inbreeding in Gyr (Bos indicus) dairy cattle
Source: BMC Genomics. 2018 Jan 9;19:34. doi: 10.1186/s12864-017-4365-3 (PMC5759835; doi:10.1186/s12864-017-4365-3)
Supplement: Supplementary file 3 — Gene Ontology (GO) terms and KEGG pathways enriched (p < 0.05) based on runs of homozygosity islands. (DOCX 30 kb) [file 12864_2017_4365_MOESM3_ESM.docx]

| Additional file 3. Gene Ontology (GO) terms and KEGG pathways enriched (p < 0.05) based on runs of homozygosity islands. | | | | | |
| --- | --- | --- | --- | --- | --- |
| **Term** | **Genes** | **P-value** | **Fold Enrichment** | **Benjamini** | **Bonferroni** |
| ***Molecular Function*** |  |  |  |  |  |
| GO:0004519~endonuclease activity | *RNASE10, RNASE1, RNASE11, RNASE2, ANG, RNASE4, ANG2, RNASE6, TSN* | 7.05E-10 | 27.09 | 1.79E-07 | 1.79E-07 |
| GO:0004540~ribonuclease activity | *RNASE10, RNASE11, RNASE2, ANG, RNASE4, ANG2, RNASE6* | 5.35E-08 | 30.98 | 6.79E-06 | 1.36E-05 |
| GO:0004890~GABA-A receptor activity | *GABRG1, GABRA2, GABRA4, GABRB1* | 2.83E-06 | 25.08 | 2.40E-04 | 7.19E-04 |
| GO:0005230~extracellular ligand-gated ion channel activity | *GABRG1, GABRA2, GABRA4, GABRB1* | 8.34E-06 | 20.52 | 5.29E-04 | 0.002115 |
| GO:0004522~ribonuclease A activity | *RNASE1, BRB* | 5.16E-04 | 75.25 | 0.025882 | 0.122886 |
| GO:0015057~thrombin receptor activity | *F2RL2, F2RL1, F2R* | 0.001023 | 56.44 | 0.042411 | 0.228966 |
| GO:0005254~chloride channel activity | *GABRA2, GABRA4, GABRB1* | 0.013623 | 7.92 | 0.392095 | 0.969320 |
| GO:0043565~sequence-specific DNA binding | *ZFHX4, SATB2, GSX2, HSF1, CEBPD, CREB1, TFCP2L1, TSN, HNF4G, MYBL1, ZNF219* | 0.017906 | 2.36 | 0.436552 | 0.989841 |
| GO:0005385~zinc ion transmembrane transporter activity | *SLC39A10, SLC39A4, SLC39A2* | 0.028209 | 11.28 | 0.554069 | 0.999302 |
| GO:0003676~nucleic acid binding | *RNASE10, RNASE1, RNASE11, RNASE2, ANG, RNASE4, ANG2, RNASE6, RNASE13, BOLL, CPSF1, BRB* | 0.028556 | 2.00 | 0.520927 | 0.999363 |
| GO:0000978~RNA polymerase II core promoter proximal region sequence-specific DNA binding | *PLAG1, HSF1, CREB1, TXK, MYBL1, STAT1, GLI2, ZNF219, CLOCK* | 0.028601 | 2.48 | 0.488324 | 0.999370 |
| GO:0004871~signal transducer activity | *PLCL1, STAT4, PTK2, IRS2, STAT1, EEF1D* | 0.030723 | 3.42 | 0.483409 | 0.999638 |
| GO:0005212~structural constituent of eye lens | *CRYGB, CRYGC, CRYGD* | 0.033714 | 10.26 | 0.488335 | 0.999835 |
| GO:0003729~mRNA binding | *SF3B1, RPL7, TSN, HNRNPC, CPSF1* | 0.044774 | 3.72 | 0.564426 | 0.999991 |
| ***Biological Process*** |  |  |  |  |  |
| GO:0007214~gamma-aminobutyric acid signaling pathway | *GABRG1, PLCL1, GABRA2, GABRA4* | 4.39E-06 | 23.09 | 0.004479 | 0.004479 |
| GO:0090501~RNA phosphodiester bond hydrolysis | *ANG, RNASE4, ANG2, RNASE6* | 2.43E-05 | 58.51 | 0.012321 | 0.024492 |
| GO:0070493~thrombin receptor signaling pathway | *F2RL2, F2RL1, IQGAP2, F2R* | 8.32E-05 | 41.79 | 0.027955 | 0.081543 |
| GO:0051482~positive regulation of cytosolic calcium ion concentration involved in phospholipase C-activating G-protein coupled signaling pathway | *F2RL2, DRD1, F2RL1, F2R* | 6.40E-04 | 22.50 | 0.150957 | 0.480341 |
| GO:0030154~cell differentiation | *EYA1, PTK2, RGS20, TRAPPC9, ANG, CREB1, ANG2, TXK, NDRG2, MYBL1, TEC* | 0.001131 | 3.52 | 0.206658 | 0.685731 |
| GO:0030335~positive regulation of cell migration | *PTK2, DRD1, IRS2, F2RL1, PDGFRA, HAS2, F2R, ATP8A1* | 0.001743 | 4.60 | 0.257174 | 0.831995 |
| GO:0060021~palate development | *SUMO1, SATB2, INSIG2, PDGFRA, ASPH, MSC* | 0.002153 | 6.55 | 0.270072 | 0.889603 |
| GO:0014068~positive regulation of phosphatidylinositol 3-kinase signaling | *PTK2, F2RL1, F2R, CD28, KDR* | 0.004684 | 7.31 | 0.451110 | 0.991760 |
| GO:0006893~Golgi to plasma membrane transport | *CHIC2, CCDC93, EXOC1* | 0.007705 | 21.94 | 0.584535 | 0.999631 |
| GO:0048146~positive regulation of fibroblast proliferation | *PDGFRA, LIG4, MYC, FN1* | 0.012713 | 8.12 | 0.729555 | 0.999997 |
| GO:0001963~synaptic transmission, dopaminergic | *DRD1, CRHBP, CRH* | 0.013002 | 16.87 | 0.703590 | 0.999998 |
| GO:0042493~response to drug | *DRD1, CREB1, TRPA1, SRP72, MYC* | 0.014415 | 5.30 | 0.709651 | 0.999999 |
| GO:0035025~positive regulation of Rho protein signal transduction | *F2RL2, F2RL1, F2R* | 0.015035 | 15.67 | 0.696085 | 0.999999 |
| GO:0006919~activation of cysteine-type endopeptidase activity involved in apoptotic process | *HSPD1, STAT1, MYC, F2R* | 0.023124 | 6.50 | 0.818759 | 1 |
| GO:0006986~response to unfolded protein | *DERL1, HSPE1, HSPD1* | 0.024397 | 12.19 | 0.814167 | 1 |
| GO:0032092~positive regulation of protein binding | *DERL1, ARHGEF7, EPB41L5, RALB* | 0.027369 | 6.09 | 0.830108 | 1 |
| GO:0001938~positive regulation of endothelial cell proliferation | *AGGF1, ANG, BMPR2, KDR* | 0.027369 | 6.09 | 0.830108 | 1 |
| GO:0010332~response to gamma radiation | *PRKDC, LIG4, MYC* | 0.032610 | 10.44 | 0.863732 | 1 |
| GO:0001662~behavioral fear response | *DRD1, PENK, USP46* | 0.035556 | 9.97 | 0.871980 | 1 |
| GO:0033077~T cell differentiation in thymus | *PRKDC, LIG4, FZD7* | 0.038600 | 9.54 | 0.879662 | 1 |
| GO:0002328~pro-B cell differentiation | *PRKDC, LIG4* | 0.040249 | 48.76 | 0.877453 | 1 |
| GO:0007030~Golgi organization | *RAB2A, ARHGEF7, CLASP1, ARFGEF1* | 0.048170 | 4.87 | 0.909521 | 1 |
| GO:0007601~visual perception | *RDH10, CRYGC, CRYGD, RPGRIP1, CNGA1* | 0.050398 | 3.58 | 0.909489 | 1 |
| ***Cellular Component*** |  |  |  |  |  |
| GO:1902711~GABA-A receptor complex | *GABRA2, GABRA4, GABRB1* | 0.001268 | 18.09 | 0.231049 | 0.231049 |
| GO:0034707~chloride channel complex | *GABRA2, GABRA4, GABRB1* | 0.005504 | 10.98 | 0.435225 | 0.681029 |
| GO:0016023~cytoplasmic, membrane-bounded vesicle | *RGS20, ANG, ANG2, SLC39A4, SLC39A2* | 0.021726 | 4.69 | 0.780342 | 0.989401 |
| GO:0000784~nuclear chromosome, telomeric region | *NABP1, PRKDC, THOC3, POLR2B, TERF1* | 0.026320 | 4.42 | 0.748508 | 0.995999 |
| GO:0005958~DNA-dependent protein kinase-DNA ligase 4 complex | *PRKDC, LIG4* | 0.038309 | 51.28 | 0.801548 | 0.999692 |
| GO:0045211~postsynaptic membrane | *GABRA2, GABRA4, GABRB1, F2R* | 0.042084 | 3.80 | 0.773124 | 0.999863 |
| GO:0097165~nuclear stress granule | *SUMO1, HSF1* | 0.050752 | 38.46 | 0.785672 | 0.999979 |
| ***KEGG pathway*** |  |  |  |  |  |
| bta04727:GABAergic synapse | *GABRG1, PLCL1, GABRA2, GABRA4, GLS, GABRB1* | 0.001070 | 5.92 | 0.184982 | 0.184982 |
| bta05032:Morphine addiction | *GABRG1, DRD1, GABRA2, GABRA4, GABRB1, PDE8B* | 0.001514 | 5.54 | 0.134743 | 0.251330 |
| bta05033:Nicotine addiction | *GABRG1, GABRA2, GABRA4, GABRB1* | 0.002333 | 8.76 | 0.138193 | 0.359928 |
| bta05200:Pathways in cancer | *COL4A2, COL4A1, KIT, STAT1, GLI2, FZD7, PTK2, CASP8, PDGFRA, RALB, TCEB1, MYC, FN1, F2R* | 0.002599 | 2.57 | 0.116858 | 0.391695 |
| bta04080:Neuroactive ligand-receptor interaction | *F2RL2, GABRG1, DRD1, GABRA2, GABRA4, HRH2, GABRB1, F2RL1, F2R, SCTR* | 0.005716 | 2.77 | 0.196679 | 0.665462 |
| bta05222:Small cell lung cancer | *COL4A2, PTK2, COL4A1, MYC, FN1* | 0.030450 | 4.18 | 0.626344 | 0.997278 |
| bta04151:PI3K-Akt signaling pathway | *COL4A2, PTK2, COL4A1, CREB1, PDGFRA, KIT, MYC, F2R, FN1, KDR* | 0.043340 | 2.12 | 0.701491 | 0.999788 |
