# Supplementary material for: Assessment of runs of homozygosity islands and estimates of genomic inbreeding in Gyr (Bos indicus) dairy cattle
Source: BMC Genomics. 2018 Jan 9;19:34. doi: 10.1186/s12864-017-4365-3 (PMC5759835; doi:10.1186/s12864-017-4365-3)
Supplement: Supplementary file 4 — Runs of homozygosity islands and signatures of selection located within or closely to those islands observed in the present study. (DOCX 19 kb) [file 12864_2017_4365_MOESM4_ESM.docx]

| Additional file 4 - Runs of homozygosity islands and signatures of selection located within or closely to those islands observed in the present study. | | | |  |
| --- | --- | --- | --- | --- |
| Author | Cattle Breed | BTA | Physical Position (bp) |  |
| **Signatures of selection** | | | |  |
| [33] | Gyr | 2 | 85,009,624 (BovineHD0200024251) |  |
|  |  | 2 | 85,010,251 (BovineHD0200029787) |  |
|  |  | 2 | 85,010,896 (BovineHD0200024249) |  |
|  |  | 2 | 103,613,639 (BovineHD0200024250) |  |
|  |  | 6 | 72,243,119 (BovineHD4100005151) |  |
|  |  | 10 | 26,147,599 (BovineHD1000008509) |  |
|  |  | 10 | 26,147,599 (BovineHD1000008509) |  |
|  |  | 10 | 26,526,068 (BovineHD1000008655) |  |
| **ROH Islands** | | | |  |
| [29] | Holstein | 2 | 87,048,075:88,162,563 |  |
|  | Polish Red | 2 | 73,788,636:74,228,539 |  |
|  |  | 2 | 81,721,478:82,654,093 |  |
|  |  | 2 | 91,411,176:92,042,146 |  |
|  |  | 6 | 75,017,253:78,259,759 |  |
|  | Limousin | 2 | 81,796,035:82,083,660 |  |
|  |  | 2 | 83,493,191:83,616,368 |  |
|  |  | 6 | 74,700,202:78,259,759 |  |
|  | Simmental | 6 | 70,704,748 72,587,575 |  |
|  |  | 6 | 81,969,240:83,256,899^1^ |  |
|  |  | 14 | 36,599,923:37,615,335 |  |
| [12,34] | Brahman, Gyr, and Nellore | 10 | 24,575,700:25,619,800^1^ |  |
| ^1^ Runs of homozygosity island located closely to those described in the present study. | | | |  |
